# Indigenous Australians Perceptions’ of Physical Activity: A Qualitative Systematic Review

**DOI:** 10.3390/ijerph15071492

**Published:** 2018-07-14

**Authors:** Emma E. Dahlberg, Sandra J. Hamilton, Fatuma Hamid, Sandra C. Thompson

**Affiliations:** Western Australian Centre for Rural Health, University of Western Australia. P.O. Box 109, Geraldton WA 6531, Australia; dahlbrg2@illinois.edu (E.E.D.); sandy.hamilton@uwa.edu.au (S.J.H.); fatuma.hamid4@gmail.com (F.H.)

**Keywords:** indigenous, aboriginal, physical activity, exercise, sport, beliefs, perceptions, systematic review, Australia

## Abstract

Given poorer health and higher rates of chronic disease seen in Indigenous populations around the world and the evidence linking exercise with health and wellbeing, recommendations for encouraging and increasing Indigenous people’s participation in physical activity are needed. This paper systematically reviews published qualitative research papers exploring issues related to the perspectives of Indigenous Australians around physical activity. Key terms relevant to attitudes, beliefs, and perceptions of Indigenous Australians on physical activity and sport were explored in 11 electronic bibliographic databases including EMBASE, Medline and Web of Science. Of the 783 studies screened, eight qualitative studies met the selection criteria; only one was exclusively undertaken in a rural setting. Four major themes emerged: family and community, culture and environment, sport, and gender differences. Men highlighted sport and going on walkabout as preferred types of physical activity while women preferred family-focused activities and activities and support for women's sport. Several studies found exercise was supported when in the context of family and community but was considered shameful when done only for oneself. Sport was regarded as playing an influential role in bringing communities together. Group, community, or family activities were desired forms of physical activity with the environment they are conducted in of high importance. These findings should inform future research and intervention programs aimed at addressing the physical activity levels of Indigenous Australians and may be relevant to other Indigenous populations.

## 1. Introduction

Physical activity is widely recognized as an important contributor to health and wellbeing [1,2,3] Evidence confirms regular exercise as an effective primary and secondary preventive measure to protect against or ameliorate chronic diseases such as obesity, cardiovascular disease, and diabetes [4]. Physical inactivity is one the six risk factors causing the most disease burden in Aboriginal and Torres Strait Islander Australians (hereafter respectfully referred to as Indigenous Australians). It accounts for 6% of the total burden of disease [5], decreasing Indigenous Australians' life expectancy by 8.4% compared to 6.6% for non-Indigenous populations [6] While the causal pathway to development of chronic diseases is complex, the importance and potential impact of increasing physical activity in Indigenous populations as part of efforts to improve Indigenous health and wellbeing warrants that this issue of physical activity be given attention [7].

Structural macrosocial factors such as colonization, discrimination, and dispossession have been identified as factors that have potentially impacted physical activity in Australia [8]. Several interventions have focused on improving physical activity at an environmental level such as the provision of community swimming pools; however, many public health initiatives are short lived and do not have the sustainability to create long lasting impacts on communities.

Many physical activity interventions and attempts to decrease the health disparities in Indigenous Australians have taken an epidemiological and quantitative approach, describing such things as time and type of physical activity each week, physical activity, and impact on measures such as weight loss, waist circumference and diabetes. There is little qualitative research complementing this data and few studies have been completed to gain knowledge of the perspectives of Indigenous Australians [9]. Yet a more in-depth understanding of Indigenous individual’s attitudes and beliefs can reveal major factors which affect physical activity. Given it is unrealistic to assume that one health intervention will have similar impacts on the whole population, research in different settings regarding the personal attitudes and beliefs that Indigenous Australians have concerning physical activity is required to inform culturally appropriate and sustainable programs/interventions to decrease the disparity in health in Australia between Indigenous and non-Indigenous Australians [10]. Identifying gaps in knowledge and understanding of what physical activity means to Indigenous Australians will also shed light on the need for such research on these dynamic topics [9].

This systematic review aims to investigate the attitudes and beliefs that Indigenous Australians have about physical activity and what physical activity means to them, recognizing that the opinions and perspectives of a target group are crucial when designing and implementing a successful program.

## 2. Methods

A systematic process was undertaken to examine literature surrounding this topic and the results were thematically analyzed to highlight major themes and outcomes. A comprehensive search of electronic databases was undertaken for the years 2000 to April 2017. The database search was supplemented by a manual search of references lists of included studies and any review articles or study protocols. The following databases were searched: Academic Search Premier, Cumulative index to Nursing and Allied Health Literature (CINAHL) Plus, Cochrane Library, Excerpta Medica database (EMBASE), JBI CONnECT, MEDLINE, PubMed, Scopus, Web of Science, AUSTIHealth, Australian Indigenous Health*InfoNet*, and Google Scholar. A combination of search terms (Aboriginal or Indigenous Australians, physical activity, exercise, sport, and attitudes or beliefs or perceptions) were used to extract information-rich data.

Studies were included if they were qualitative in design, published in peer-reviewed journals, based in Australia, included Indigenous Australians, and were written in English. Studies were excluded if they were systematic reviews, protocols, conference abstracts or with non-Indigenous populations.

Titles and abstracts of identified studies were screened for eligibility by three researchers and the duplicates were removed. Eligible studies were examined using a systematic review template which was adapted from the Joanna Briggs Institute Reviewer’s Manual [10]. The articles were read and re-read to gain the greatest understanding possible and obtain an accurate review of the data. Study demographics were recorded, including the processes for participant selection, and the population involved. Information on the study year, study aim, and key words were also identified. A template based upon the Joanna Briggs Institute data extraction tool for systematic reviews was used for data extraction, following the methods used in the Reviewer's Manual [10]. The qualitative methods used were critically reviewed to assess the effectiveness and appropriateness of the data collection of each of the selected studies. The effectiveness of the studies was evaluated using the Critical Appraisal Skills Programs (CASP) tool while the appropriateness of the included studies referred to what the qualitative data derived from the study reveals about Indigenous Australians perceptions of physical activity.

Three reviewers also appraised the included studies for quality and risk of bias utilizing the CASP [11]. This involved a series of ten questions that assessed the appropriateness of the study research design, sampling methods, data collection methods, ethical issues, data analysis, findings, and the value of the research. When using the CASP tool, the researchers' relationship with their participants is considered and any potential biases identified.

## 3. Results

**Figure 1:**
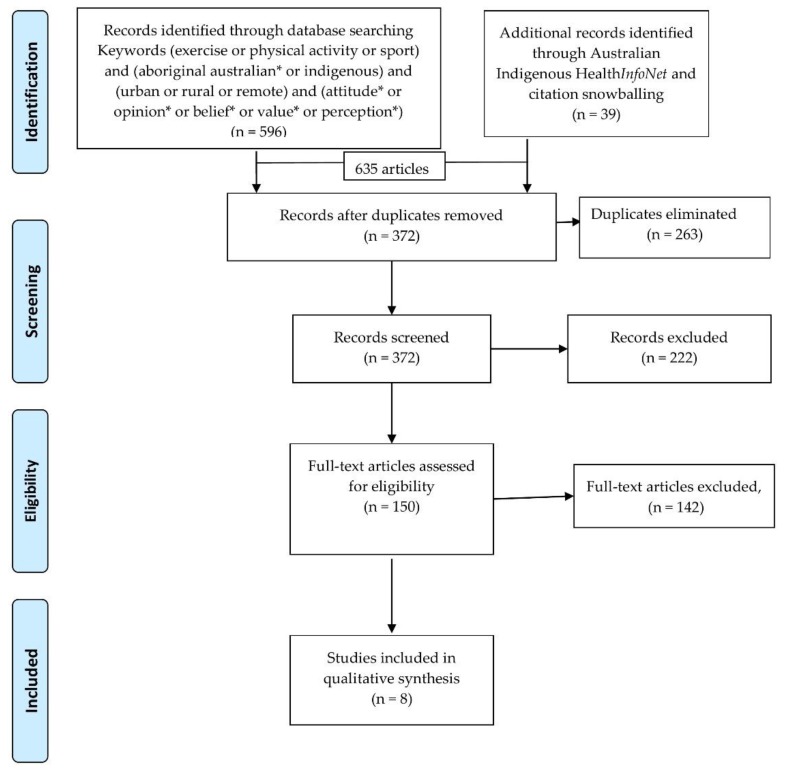
PRISMA Flow diagram showing systematic approach to literature search [12].

The flow diagram outlining the process of screening and selection of articles is shown in Figure 1 [12]. Of the 635 articles identified, most were identified in library database searches (596) with a further 39 were found through the Australian Indigenous Health*InfoNet* or citation snowballing in which promising references in footnotes and bibliographies of articles were identified and examined for relevance [11] There were 263 duplicates recorded and removed. From the 372 potential articles, 222 were initially excluded based upon screening of abstracts as not relevant to the research question, often because they were quantitative and assessed numeric data from physical activity programs. After the final 150 articles full text articles were screened, 142 were excluded for not having a focus on qualitative information or on Indigenous Australians to be relevant to our study aim. Eight articles met the eligibility criteria and were subjected to analysis of themes and assessment of quality, following the methods stated above. All eight of the studies performed well in terms of qualitative rigor on the CASP assessment; one only met eight of the ten CASP requirements [13].

Two articles provided valuable content regarding Indigenous Australians’ perspectives on physical activity, but did not specifically meet the selection criteria and thus were not included in the final synthesis [14,15]. All eight studies were conducted in Australia, in various types of environments between the years of 2000 and 2017 and targeted at only Indigenous Australians.

Each study utilized a similar qualitative process using a combination of semi-structured interviews, focus groups, demographic questionnaires, and surveys*.* One study utilized drawings and images to provide stimuli for face-to-face interviews [8] where as other studies used a semi-structured interviewer guide to direct interviews. Seven of the studies took into consideration cultural biases within their frameworks with all but one of the studies using Indigenous researchers or assistants to meet cultural needs of the participants [13]. Three studies were conducted in both urban and rural environments [16,17,18], four in urban [13,19,20,21], and one in a rural environment [8] (Table 1). 

The major themes recorded are representative of answers to questions such as, “what type of physical activity do you do?”, “what made it difficult for them” [21], “what influences your physical activity?” [21], “how physical activity is viewed within a community?”[13] and “how would you describe physical activity?”[8]. Articles addressed topics including perspectives of physical activity, what influenced participation, how it was viewed in the community, and barriers to participating in physical activity *(*Table 2). There were four distinct themes identified although they had areas of overlap and intersection, with each theme identified across multiple articles included in the review.

### 3.1. Family/Community

The connection to family and community is consistently reported as very important in Indigenous cultures [21]. Several studies found that family determines a child’s level of activity and type of sport they practice [19,20]. Children rely on their parents as a means of transportation to sport trainings and games as well as financially for gear and club fees [20]. Nelson et al. assessed Indigenous youths’ perceptions of physical activity and found that family was identified to be at the forefront of their motivation in the type of sport they decide to play and how they connect with family members [19]. Two participants from difficult family backgrounds reported that they had used physical activity to gain independence and get away from home, but they did not enjoy exercise as much as the other participants [20]. Overall, children viewed their family as having a large impact to their health and wellbeing [20].

Many factors linked to family affected Indigenous Australians’ participation in physical activity. Overall, physical activity is embedded in a complex web of meaning which links family and their larger Indigenous community together. When exercise was done solely for the individual, it evoked the notion of shame [13,16]. Individuals can feel shamed if participating in exercise done only for personal reasons, as this comes from notion that they are doing something for themselves in isolation of their family and community. However, when a person is ill, or the exercise was done to improve their health, it was considered not to evoke shame. Thus, physical activity was perceived positively as a form of secondary or tertiary prevention but viewed negatively if done as a form of primary prevention [21]. The strong social connections with family, culture and community are far more important than any personal health benefit which solitary exercise may give someone [13]. It is important to note that this notion of shame is more prominent in Indigenous females than males, particularly because the females play an important role in the family [18].

The men interviewed in the study of Mellor et al. discussed the importance of family and physical activity [17]. Some men stated that their reasons for being healthy and physically active stemmed from people in their family and community having health problems, and that they want to be fit to avoid similar health troubles. They also indicated that there was a lack of role models that practice positive health behaviors such as exercising frequently [17]. The physical activity practices seen within a family can clearly impact each family member’s attitudes and beliefs about physical activity. MacDonald and colleagues found that parents expressed the importance of their children being active and that they would benefit from having family sports and more facilities in the area [16]. The importance of family and the parents’ desire for their children to be active reinforces the idea of promoting physical activity at the family level.

### 3.2. Culture and Environment

Culture and environment proved to be a significant theme across all studies. Information in this topic includes information that references the relationship with the land, the environment of where the physical activity is performed and how that environment impacted participant attitudes about the activity. Indigenous cultures within Australia place a large emphasis on connection to and appreciation of the land. Prior to colonization, Indigenous Australians incorporated physical activity within their everyday lives [22]. The connection to the land holds great importance to individuals living in the rural and remote areas of Australia [13].

In the study conducted in remote areas of Australia, Thompson et al. found a strong connection between physical activity and the land [8]. Indigenous people tended to associate physical activity most closely with “work” and “walkabout”. The specific purpose of this type of physical activity related to the consumption and distribution of natural and cultural resources including the acquisition of food, fire wood, and other essential resources they utilize [8]. Mellor and colleagues reported similar results with participants feeling that going bush was not only a form of physical activity they enjoyed, but one which benefited their mental and social health. They also felt that more Indigenous specific sporting facilities and activities would encourage participation [17].

Specifically, participants expressed different cultural preferences for the way physical activity was organized and where and when it occurred [8,18]. Thompson et al. and Stronach and colleagues noted how the type of activity as well as the time and place it occurred strongly influenced Indigenous peoples’ participation in physical activity. For example, many participants preferred to go swimming in their local billabong rather than in local government funded swimming pools. It was more comfortable for them to do things their way in the bush rather than the European way. Overall, there was strong evidence around idea that people are more active and comfortable in their own cultural spaces. It is important to note that the physical activity carried out in the bush is different to that carried out within the community [8]. These findings suggest that rural Indigenous Australians consider exercise as an activity performed within their own environment which in turn connects them with their culture and community.

In their study, Thompson and colleagues also reported there was strong evidence of success in promoting physical activity that has a physical connection with their natural environment. Natural and cultural resource management and associated activities which occur “on country” were identified as preferred Indigenous health promoting activities. Both male and female participants spoke of the bush as a “healthy place”. The activities conducted “on country” were viewed as educational opportunities to facilitate the transfer of knowledge to younger generations [8]. Living in an urban environment may result in a less active lifestyle as opposed to those who lived in rural and remote areas where individuals worked on the land and engaged with their cultural background [8].

According to Thompson and co-authors, the environment is considered a large barrier for physical activity in urban settings. There was evidence of a decline in physical activity among Indigenous participants who moved from a rural or remote area into an urban setting [8]. Hunt et al. found that the social environment placed a constriction on physical activity as many participants felt they would be judged for participating in exercise in the public or walking down the street [21]. Female participants in particular expressed notions of security worries and not feeling safe when exercising in public. The theme of a “safe place” was also identified in the study of Nelson and colleagues, where participants aged 11–13 years spoke of wanting “safe places” that encouraged connections with family, friends and community to increase their activity levels [20]. Therefore, Indigenous Australians place a big emphasis on their physical and social environment when it comes to physical activity.

### 3.3. Sport

Sport was another common theme which emerged from most studies. Data on the impact of sport participation as a form of physical activity on individuals’ lives, interpersonal relationships, and the impact sport has on attitudes toward physical activity were all discussed. Sport was a favored form of physical activity for many young people as it involved competition and being a part of a team. Winning tended to provide a form of motivation for younger Indigenous individuals as they perceived it as getting a reward for doing sport. There was also evidence of the positive impact in the strong sense of collective identity and pride that is experienced when part of a team [19]. Being a part of individual and team sports is an important part of Indigenous people’s lives [13].

According to Stronach and colleagues, participating in sport keeps people connected to family and community. Whether it is assisting family members in sport or parents serving as role models to their children, social bonds formed through sport are essential in building positive relationships [17,18]. Nelson et al. reported that many sporting and recreational activities were also addressed within the context of family, community, and culture [19]. Particularly with participation in team sports, family tended to have a strong influence on the type of sport individuals played as well as reinforcing ties that bind individuals to their communities. Sport such as backyard cricket, football, and rugby were often played when visiting or meeting family [21]. This reinforces the theme of sport and physical activity being a positive occurrence when undertaken within the context of family and wider community.

Adults in Mellor and colleagues study reported that there should be a larger variety of sports shown to kids [17]. There are many ways to be physically active and participating in a variety of sports was seen to spark their interest and promote an active lifestyle [17], and can also open doors for the child’s future. Expanding their skill sets in sport will help set them up for success [18]. The theme of sport as a saviour or future for many young people was identified in the Nelson et al. study [19]. Many young Indigenous Australians aspire to be elite athletes, not only for financial gain but also for the love of the activity [23]. When involved with sport at a high level, many young people in this study also felt there was a responsibility to provide a role model for other Indigenous youths. They viewed their Indigenous background as “black magic” and that it improved their athleticism. Being involved in sport also tended to keep young people active and reduced boredom [19]. Nelson and colleagues also found that when kids played sports, it caused them to think about other aspects of their health [24]. Sport was also a good way to foster a child’s confidence and knowledge of their health and physical activity.

This theme in particular was largely dominated by males who viewed sport as a main form of physical activity. Males also tended to focus more on competitive contact sports such as football, rugby, and boxing [21]. Women reported taking on an administrative role with sporting events in the community rather that participating in the events [16]. Parents expressed the need for there to be more sport clinics for girls and scholarships for Indigenous women to encourage their participation [18].

A lack of resources was also addressed in Hunt et al. Participants discussed perceived affordability of some physical activity programs as well as a lack of sustainability of many physical activity health promotion programs that target this population. Many participants desired an ongoing sporting team or club that would continue and not be short lived [21].

### 3.4. Gender

According to many of the studies, gender played a key role in defining Indigenous Australians’ attitudes and beliefs regarding physical activity with various motivations and attitudes toward physical activity separated from the male and female perspective. For males, physical activity was very sport dominated. When asked what they believed was physical activity, they stated that it was, “any type of physical activity that gets the heart rate up…fitness…weights…and/or sport” [21]. Many men also felt that being an Indigenous male increased their health risks, thus giving them incentive to be more physically active [17].

In Indigenous culture, the females have a large responsibility to family. When asked about their views on physical activity, they stated, “household chores, laundry, not necessarily sport but mainly anything physical” [21]. According to Stronach et al., Indigenous women have lower rates of participation in sport compared to non-Indigenous women. In fact, many women find it hard to choose physical activity because they feel shame and guilt around being involved in activities which only benefit the individual rather than the family or community [18]. Similarly, MacDonald and colleagues found that many women felt that they are not able to play sport because the men do not approve or because the men are already busy with sport and someone needs to take care of the children [16].

A person's gender also influenced participation on physical activity, with differing barriers reported by men and women [21]. Young Indigenous women frequently described feelings of shame or embarrassment when being physically active [14]. For males, a commonly occurring barrier was ongoing injury, disability, or illness which constrained them from participating in physical activity [21]. Men also stated that their experience with racism in sport had prevented them from playing [17].

## 4. Discussion

This review adds to the qualitative research on the complex perspectives of Indigenous Australians regarding physical activity. It is important to capture the views and knowledge of the population before implementing programs. Factors that define a culture also contribute to various health behaviors such as roles in a family, community responsibility, and an individuals’ values and priorities [21]. As identified in this review, Indigenous culture impacts on physical activity [8,13] with history and connection to the land, gender roles, and access to facilities in rural and urban environments all contributing to the types of activities that Indigenous Australians prefer [17,20,21]. The setting and environment of the physical activity was an important factor influencing Indigenous Australians' willingness to participate. There were differences to modern Western practices of individualized physical activity which should be considered when developing exercise promotion programs that are culturally relevant.

The results highlight the differences in perspectives according to gender. Indigenous females have lower rates of participation in sport and physical activity and after gathering their perspectives, programs can be adjusted to alleviate perceived barriers [25]. Females do not feel that they have similar freedoms to partake in physical activity as men. Their responsibility to the family, home, and the concept of shame limits their participation [16]. Shame is a concept in Indigenous culture where one feels singled out from the group, positively or negatively, and thus loses the trust and security gained from the group [26]. Although shame is experienced by all genders, shame proves to be one of the largest hindrances to young women actively participating in physical activity [14] and hence this issue should be addressed when targeting this population.

Sport is viewed as a form of physical activity that touches every age and gender. It is an outlet for success and can create valuable Indigenous role models in families and communities. Many participants in the studies reported believed that being Indigenous improves their athletic ability, and it is already a major aspect of Indigenous men’s life [19]. Young women were identified as a population which requires specific attention when developing sporting programs. With their feelings of shame and lack of confidence, there is a desire to implement more sport clinics for young women and also to create scholarships targeting Indigenous women to encourage their participation [18].

According to the Australian Bureau of Statistics, Indigenous men had a 15% higher participation rate in physical activities than Indigenous women. With age, participation decreased in both genders [27]. Many older participants in the included studies revealed that they are not as active as they were in their youth, and there are limited healthy older role models for children [17,18]. This raises the question of what helps maintain physical activity in Indigenous Australians throughout their lifetime. There is a desire for family-oriented physical activity and sport because it can positively bring the family and community together [19] and the younger population can inspire and motivate older Indigenous individuals. To ensure appropriateness, interventions targeting this population group should take these preferences into consideration.

The environment plays a large role in the barriers of participation and should be addressed when developing a program. The expense of clubs and programs is an issue for some. In rural areas, individuals stated that the climate, lack of equipment, and the lack of sustainable programs also makes it difficult to be physically active [14]. Many also stated that the transition from rural areas to the city impacts on their physical activity levels because that connection with the land is lost [17]. Only one of the included studies was performed in a remote area, two were undertaken in rural and urban settings, and four solely explored views in urban areas, yet the differences in perspectives were not identifiably different. The preferences for types of activities was similar, with a consistent appreciation for the natural land [8]. The land is important to the Indigenous culture and the existing literature emphasizes the success of ranger programs and land care as physical activity promotion [22].

Despite the high quality of the included studies, limitations exist for this review. Many studies did not list the specific questions that were asked in collecting their qualitative data. The common themes may have come about based on the type of questions that were asked, rather than arising from open-ended exploration of attitudes and beliefs. However, studies addressed cultural sensitivity in their methods, with only one study not using an Indigenous researcher and all acknowledged the potential for bias [19]. Physical activity is a broad concept and means different things to different people, with definitions often more physiological in that they refer to any bodily movement provided by skeletal muscle that requires energy expenditure [28]. The included articles did not define what they deemed as physical activity, allowing participants to express their own definition which ranged from walking and going bush to organized or competitive sport. This makes it difficult to compare views across genders and age groups. More specific framing would be useful in future research.

Areas which have previously been identified as needing further examination are whether programs that show promising results in the short term are effective in the longer term (over five years), the effectiveness of sport promotion programs in increasing participation in sport among Indigenous children and adults, and examining what strategies are effective in promoting participation in sport by older men, and adult and older women [24]. Given that we identified relatively little literature on the views of Indigenous Australians regarding sport and the dearth of studies from rural and remote areas in particular, further research across Australia from these settings is needed. More research is required into the attitudes, perceptions, and beliefs that Indigenous Australians’ have surrounding physical activity as it is unrealistic to assume that one health intervention will work the same way for the whole population. To decrease the gap in health disparities of Indigenous Australians, this review can inform the development of programs or interventions that are culturally appropriate [29] which may improve their sustainability. Increasing understanding of what physical activity means to Indigenous Australians sheds light on the need for research on these dynamic topics and the design of effective interventions [9].

## 5. Conclusions

This review shows that there is considerable diversity in the meanings identified in studies of Indigenous Australians around meanings and perceptions surrounding physical activity. The information and themes drawn from the literature have provided answers to the question of what attitudes and beliefs Indigenous people have toward physical activity. The main themes emerging from the literature include family and community, culture and environment, and sport with gender differences and several barriers to participation also identified.

Physical activity was viewed in three ways: exercise, everyday activities, and sport [13]. Exercise was seen as a relatively disconnecting experience unless done with family and friends whereas everyday activities were a necessary part of Indigenous people's lives. Most people discussed moving around, being active, and being mobile to describe their everyday activities [13]. There was strong evidence for this point in many of the articles.

The Western concept of individual exercise programs would appear not to be the best approach when promoting physical activities in this population. Group, community, or family activities are all approved forms of physical activity. High importance is attached to the type of environment in which exercise is undertaken, with the natural land remaining influential in Indigenous culture. Particular barriers to participation exist for specific groups such as young women and adults revealed and should be considered in future program design. 

Overall, there was a limited amount of literature on the topic with the information from the identified literature in no way representing the Indigenous population as a whole, but instead providing an indication into viewpoints of Indigenous individuals and communities. The evidence can assist with identifying future research needs for this population. Understanding the perceptions and opinions of target groups is important when implementing interventions to encourage physical activity, improve wellbeing and reduce the risk of chronic disease if efforts are to be effective and sustainable. These findings should inform future research and intervention programs aimed at addressing the physical activity levels of Indigenous Australians and may be relevant to other Indigenous populations.

## Figures and Tables

**Table 1:** Description of included studies.

First Author Year Published Location	Aim	Participants	Methods	Outcomes
Thompson 2013 Rural	To provide perspectives on physical activity as seen by Indigenous people living remotely in the NT	23 Indigenous from two remote communities in NT aged between 16 and 25 years	Semistructured interviews, recorded observations of community, interviews transcribed externally and reviewed	PA associated with the land, activity time and place important, interaction with natural environment highly valued
Macdonald et al., 2012 Rural and Urban	Qualitative data that gauged opinions on health, preferred types and barriers of physical activity taken from a larger study called “Reversing the Trend”	21 Indigenous families from Far North Queensland	Semistructured and open interviews held by Indigenous researchers, transcribed and major themes (shame, gender roles, deferral of responsibility) extracted	Large difference between gender and PA attitudes, PA is supported when it is in the context of family and community but not individually
Mellor et al., 2016 Rural and Urban	To identify Indigenous men's beliefs about physical health using the Health Belief Model	150 men ages 18–35 years from Broome, Mildura, and Melbourne	Participatory action research framework was used; Stage 1: focus groups; Stage 2: semistructured interviews followed by 20 additional interviews; transcribed and coded into themes	Men felt activity on the land benefited health; racism in sport, financial issues, and transport were barriers to participating
Stronach 2016 Rural and Urban	Explore the meaning and experience of sport and physical activity to Indigenous Australian women and how it contributes to their health	22 Indigenous women from Redfern NSW and Flinders Island, TAS	Indigenous researcher, one-hour conversations in groups or individually; Dadirri method; data transcribed and coded themes and discourses using Critical Race Theory	Indigenous cultural beliefs, traditions, historical factors, gender factors and geography impact PA; see PA as a positive influence in their life
Nelson 2009 Urban	To discover urban Indigenous youth perceptions of sport and physical activity and how it influences their lives	14 Indigenous youth aged 11–13 years attending independent urban schools, eight girls and six boys	Indigenous researcher; participant interviewed six to eight times over 2.5 years; data analyzed using Critical Discourse Analysis	Sport and PA as a connection with friends, family, and community
Nelson 2012 Urban	Discover the ways that urban Australian Indigenous young people perceive and articulate the risks associated with their health and physical activity	Eight girls and six boys aged 11–13 years who attended an urban school in a major Australian city	Indigenous researcher, participant interviewed six to eight times over 2.5 years, perception of risk analyzed using Critical Discourse Analysis	Sport creates awareness of health, desire to be active but not "over-do" it, family impacts activity levels with encouragement and transport, do not see being Indigenous as an impact on health
Thompson 2000 Urban	Develop an appropriate epidemiological risk factor survey instrument for Indigenous people living in Melbourne, Victoria. Determine how exercises or being active, is important not only for prevention of diabetes but also for CVD and other chronic disease	In-depth interviews—38 Indigenous adults aged 20–50 years with and without diabetes; Group Discussion—19 with and without diabetes	3 Phases: (1) Ethnographic mix of qualitative methods including in-depth interviews, focus groups and participant observation. (2) Development of a questionnaire (3) Pilot case control study	View PA in 3 ways: everyday activities, exercise, sport; sense of shame if someone is doing sport for their own personal gain
Hunt et al. 2008 Urban	Determine the meaning, barriers, and strategies to promote physical activity among urban Indigenous Australians	96 Indigenous adults living in Brisbane, Queensland. First round: 20 males, 14 females Second round: 25 males, 37 females	First round: 6 focus groups Second round: 11 focus groups Transcribed and thematically analyzed into three main topics	Females: anything physical, house work; Males: sport dominated Walking and family-oriented activities were stated as common activities for both genders. Barriers: environment, safety, affordability

Abbreviations: PA = physical activity; NT = Northern Territory; NSW = New South Wales; CVD = cardiovascular disease.

**Table 2:** Major themes related to physical activity compared across studies

First Author Year	Major Themes			
	Family/Community	Culture/Environment	Sport	Gender
Thompson 2013	Not discussed	Work and walkabout: strong associations with physical activity and the land, interaction with natural environment highly valued	Not discussed	Women: preference to be physically active their own way in the bush
Macdonald 2012	Physical activity is supported when in the context of family and community but not as an individual	Little continuity of programs	Women talking of sport focused on men and boy participation	Women feel men have more freedom to be physically active
Mellor 2016	Lack of healthy family role models	Men from rural areas found activity on the land to benefit their health	Greater variety should be shown to kids	See being an Indigenous male as a risk to their health
Stronach 2016	Women accept responsibility for children and family, serve as role model, develop social bonds	Sport benefits community prevent engagement of unsafe behaviors, pathway out of poverty	Promotes equality, desire for more sport clinics and scholarships for girls/women	Prefer attending Indigenous women only classes, “safe place”
Nelson 2009	Sport used as a connection to family, family has influence on sport participation	Indigenous background perceived to improve athleticism	Used to prevent troubled behaviors, create a future career for themselves	Not discussed
Nelson 2012	Family impacts activity levels with encouragement and transport	Do not think Indigenous status impacts their health	Sport increases health awareness, desired to be active, want more Indigenous people in sport	Not discussed
Thompson 2000	Exercise is positively supported in the context of family and community	Participation in sport brings together the community, exercise for personal improvement is seen as shameful	Individual and team sports are an important part of Indigenous life	Men state working would make them more physically active
Hunt 2008	Family orientated activities, especially for women	Desire to have group-based activities in a fun environment	Men: football, boxing, and touch football. Women: netball basketball and touch football	Gender specific perceptions of physical activity

## Appendix A. Examples of Systematic Review Search (April 2017)

Examples of the search terms used for each database and number of articles found are shown in Appendix A.

**Search Terms**	**Number of Citations**
**Medline**	
(exercise or physical activity or sport).mp. (mp = title, abstract, original title, name of substance word, subject heading word, keyword heading word, protocol supplementary concept word, rare disease supplementary concept word, unique identifier)	288,673
(aboriginal australian* or indigenous).mp. (mp = title, abstract, original title, name of substance word, subject heading word, keyword heading word, protocol supplementary concept word, rare disease supplementary concept word, unique identifier)	22,459
(urban or rural or remote).mp. (mp = title, abstract, original title, name of substance word, subject heading word, keyword heading word, protocol supplementary concept word, rare disease supplementary concept word, unique identifier)	245,761
(attitude* or opinion* or belief* or value* or perception*).mp. (mp = title, abstract, original title, name of substance word, subject heading word, keyword heading word, protocol supplementary concept word, rare disease supplementary concept word, unique identifier)	2,061,558
All combined with AND	24
**CINAHL Plus**	
physical activity or exercise or sport	173,138
aboriginal australian* or indigenous	8003
urban or rural or remote	82,188
attitude* or opinion* or belief* or value* or perception*	555,228
All combined with AND	21
COCHRANE LIBRARY	
MeSH (motor activity) explode all trees	20,344
MeSH (exercise) explode all trees	17,404
MeSH (sports) explode all trees	12,132
Aboriginal Australian* or indigenous	628
Urban or rural or remote	13,955
Attitude* or opinion* or belief* or value* or perception*	153,929
Combined with AND	1
**EMBASE**	
(exercise or physical activity or sport).mp. (mp = title, abstract, heading word, drug trade name, original title, device manufacturer, drug manufacturer, device trade name, keyword, floating subheading)limit 1 to yr = “1990–Current”	481,800
(attitude* or opinion* or belief* or value* or perception*).mp. (mp = title, abstract, heading word, drug trade name, original title, device manufacturer, drug manufacturer, device trade name, keyword, floating subheading)limit 3 to yr = “1990–Current”	2,500,154
(aboriginal australian* or indigenous).mp. (mp = title, abstract, heading word, drug trade name, original title, device manufacturer, drug manufacturer, device trade name, keyword, floating subheading)limit 6 to yr = “1990–Current”	27,984
(urban or rural or remote).mp. (mp = title, abstract, heading word, drug trade name, original title, device manufacturer, drug manufacturer, device trade name, keyword, floating subheading) limit 8 to yr = “1990–Current”	281,361
All combined with AND	30
Note: Words were slightly modified for different databases.	
